# An Updated Review of Toxicity Effect of the Rare Earth Elements (REEs) on Aquatic Organisms

**DOI:** 10.3390/ani10091663

**Published:** 2020-09-16

**Authors:** Nemi Malhotra, Hua-Shu Hsu, Sung-Tzu Liang, Marri Jmelou M. Roldan, Jiann-Shing Lee, Tzong-Rong Ger, Chung-Der Hsiao

**Affiliations:** 1 Department of Biomedical Engineering, Chung Yuan Christian University, Chung-Li 320314, Taiwan; nemi.malhotra@gmail.com; 2 Department of Applied Physics, National Pingtung University, Pingtung 900391, Taiwan; hshsu@mail.nptu.edu.tw; 3 Department of Bioscience Technology, Chung Yuan Christian University, Chung-Li 320314, Taiwan; stliang3@gmail.com; 4 Faculty of Pharmacy and The Graduate School, University of Santo Tomas, Manila 1008, Philippines; mmroldan@ust.edu.ph; 5 Center for Nanotechnology, Chung Yuan Christian University, Chung-Li 320314, Taiwan; 6 Department of Chemistry, Chung Yuan Christian University, Chung-Li 320314, Taiwan

**Keywords:** rare earth elements, ecotoxicity, aquatic biota, bioavailability, toxicity indicator

## Abstract

**Simple Summary:**

Rare earth elements (REEs) have become important metals in modern-day technology. The process of discarding obsolete equipment containing REEs, use of REEs containing phosphate fertilizers, mining, and dispersion from indigenous rocks may increase the likelihood of REEs contamination in water bodies. Thus, the contamination may contribute to their release into surrounding ecosystems. This review paper aims to understand the bioavailability, accumulation, interaction, and toxicity criteria of REEs with aquatic organisms. The currently available literature is confined to reports of a few REEs. There exists substantial knowledge gaps persistence on the health effects. The REEs related to health effects also point to favorable and adverse effects after exposure. The studies have also demonstrated that REEs follow a hormetic concentration- related trend, making it stimulatory or protective at low dose levels and adverse at high dose concentrations. Based on limited information on REEs’ effects, we highlight the need for more detailed studies on REEs’ effects individually/collectively. The studies should also include detailed outcomes related to growth inhibition, embryotoxicity, cytogenetic effects, and organ-specific toxicity. We believe that aquatic biota is an efficient platform to study the effects of REEs and might yield beneficial human health information.

**Abstract:**

Rare earth elements (REEs) or “technology metals” were coined by the U.S. Department of Energy, a group of seventeen elements found in the Earth’s crust. These chemical elements are vital and irreplaceable to the world of technology owing to their unique physical, chemical, and light-emitting properties, all of which are beneficial in modern healthcare, telecommunication, and defense. Rare earth elements are relatively abundant in Earth’s crust, with critical qualities to the device performance. The reuse and recycling of rare earth elements through different technologies can minimize impacts on the environment; however, there is insufficient data about their biological, bioaccumulation, and health effects. The increasing usage of rare earth elements has raised concern about environmental toxicity, which may further cause harmful effects on human health. The study aims to review the toxicity analysis of these rare earth elements concerning aquatic biota, considering it to be the sensitive indicator of the environment. Based on the limited reports of REE effects, the review highlights the need for more detailed studies on the hormetic effects of REEs. Aquatic biota is a cheap, robust, and efficient platform to study REEs’ toxicity, mobility of REEs, and biomagnification in water bodies. REEs’ diverse effects on aquatic life forms have been observed due to the lack of safety limits and extensive use in the various sectors. In accordance with the available data, we have put in efforts to compile all the relevant research results in this paper related to the topic “toxicity effect of REEs on aquatic life”.

## 1. Introduction

The discovery of rare earth elements (REEs) started at the end of the 18th Century. The REEs include the 15 lanthanide elements, and the Yttrium and Scandium. Lanthanides are a group of fifteen elements of atomic number 57 through 71, including Lanthanum (La), Cerium (Ce), Praseodymium (Pr), Neodymium (Nd), Promethium (Pm), Samarium (Sm), Europium (Eu), Gadolinium (Gd), Terbium (Tb), Dysprosium (Dy), Holmium (Ho), Erbium (Er), Thulium (Tm), Ytterbium (Yb), and Lutetium (Lu). The REEs of lanthanides exhibit similar properties throughout the series with seven f-orbitals in the f subshell, accommodating two electrons each orbital. It results in fifteen possibilities of filling the f subshell, and thus, displays the similarities in physical and chemical properties and small differences in atomic weight across the series. They are usually silvery-white metals in appearance and possess high electrical conductivity. According to the United States Geological Survey (USGS), REEs are relatively abundant in the Earth’s crust, but are widely dispersed [1,2,3,4]. The term “rare” in rare earth metals may have derived from the difficulty of obtaining them as purely individual elements because the chemical differences between individual lanthanides are small. Though recently, many technologies have emerged to yield high-quality REEs [5]. Several techniques have been used to extract and purify REEs, such as ion-exchange, chemical precipitation, liquid-liquid extraction, and adsorption [6,7].

REEs are typically classified into two categories, namely, light rare earth elements (LREEs) (Ce-Sm plus Y) and heavy rare earth elements (HREEs) (Eu-Lu) [7]. According to the data, 110 million tons of REEs reserves with valuable deposits are scattered all over our planet [8]. The 97 percent of the global REEs supply is produced by China [9], 1.73 percent by Russia, and 1.18 percent by the USA (some minimum other reserves in Brazil and the Democratic Republic of Congo). Many REEs have few effective substitutes and low recycling rates, which has raised many questions about their future supply [8,9,10]. The REEs are mainly available in six mineral resources, namely, bastnaesite, monazite, xenotime [5,11,12], loparite [11,13], apatite, and ion-adsorption clays [11,14]. Usually, REEs are found in phosphates, carbonates, fluorides, and silicates [15]. The process and separation of REEs are carried out by very few companies worldwide [9]. REEs have widespread applications in various industries [15]. All the industrial applications of REEs are compiled in Table 1.

The extensive usage of REEs in different industries may eventually lead to severe contamination of the surrounding environment, agriculture, aquatic fauna, and soil, which might, in turn, cause severe damage to human health. Different studies were conducted to evaluate REEs’ toxicity effect in soil, water, model organisms, and humans working in close collaboration with the REEs field mentioned in recent times [16,17,18]. Many research groups are studying the toxicity effects of different REEs due to REEs’ persistence in the environment for a long time. A group of researchers highlighted the use of local Moran’s I test in assessing the spatial statistical analysis of REEs contamination, especially in the hotspots of urban soil. They indicated that REEs enrichment in urban soils might occur due to the complex metal recycling sources, waste disposal, agricultural practices, and vehicular emission [19,20,21,22,23]. Moreover, a study conducted on the water samples collected from ~500 stream waters and associated sediments over ~400 km^2^ region declared that the rare earth elements (REE + Y) could be divided into three different pools (dissolved fractions, labile fractions, and detrital fraction), where the REE+Y partitioning into the sediment phase has been attributed to adsorption or precipitation, with Eu as the most mobile of REEs and δ-MnO_2_ as a dominant sink for Ce in comparison to other REEs [24]. All of these studies correspond to the fact that REEs’ concentrations depend on pH, weathering, redox conditions, and types of clay minerals in surroundings, complexation of REEs with organic and inorganic ligands, and the relationship with the environment. Therefore, it is essential to perform more studies on different environmental levels and organism levels to understand better REEs’ functioning in establishing the safety-based limits accordingly [25,26].

REEs have also been known for their hormetic effect. The window is now open to the detailed conceptualization of molecular biology, pharmacology, toxicology, and risk assessment other than environmental risk assessment. The literature from animal studies and finite data from human occupational exposures proposes REEs-induced tissue-specific bioaccumulation and liver, lungs, and brain damage. The adverse outcomes might be enhanced following combined exposure to REEs and acid pollutants [27,28].

In a study, Ce (IV) and La (III) were exposed to sea urchin (embryos and sperms) for 72 h and 1 h, respectively, at a concentration range of 10^−8^ to 10^−5^ M. The results depicted 100% mortality at an exposure concentration of 10^−5^ M of Ce (IV), and 100% developmental defects at an exposure concentration of 10^−5^ M of La (III) with no mortality. The sperm exposure of both Ce (IV) and La (III) induced a decline in sperm fertilization success at the highest tested concentration (10^−5^ M) [29]. The different concentration-response relationships demonstrate the knowledge gaps of REEs’ effects and should be worked on in the forthcoming studies.

The REEs’ exposure has been differentiated into the suspected and occupational routes. They can be exposed to some useful electronic material/device (or REEs dust in mining for that matter have proven to be harmful) or through other lesser-known channels still undercover. For example, Gadolinium (Gd) used in Magnetic Resonance Imaging (MRI) as a contrast agent has been reported to cause renal toxicity. It was shown that patients with an orderly renal system could clear gadolinium-chelate complexes quickly out of the system. However, in the case of patients with an impaired renal system, the gadolinium-chelate complex’s clearance is not efficient, and this extended stay of the gadolinium-chelate complex might allow the dissociation of Gd from chelate and then bind to tissue. Such cases have been observed through skin biopsies [30,31] and other tissues of nephrogenic systemic fibrosis (NSF) patients [32,33]. On equal terms, a study highlighted the high risk of acute kidney injury in a population group under the usual dose of MRI, 16.5% of the tested population developed acute kidney disease with no significant difference in age, gender, or baseline glomerular filtration rate (GFR) [34,35].

In this review, we present the importance of the REEs’ speciation in water bodies and some toxicity insights caused by selective REEs in aquatic life forms. As water bodies are in contact with the environment, soil, waste materials, and life form, they provide an excellent, sustainable platform to study REEs’ toxicity effects.

## 2. Speciation of REEs in Water Bodies

REEs’ chemical speciation plays a vital role from an environmental point of view. REEs are close in physical and chemical properties; they indicate the specific molecular form under which the element might get transported in a hydro system in a more complex manner. These elements can form complexes with cation, anion, neutral ion, or ligand (organic, inorganic). In a complex, REEs vary between chemical forms and are affected by surrounding conditions such as pH, salinity and weathering conditions; thus, it is challenging to measure individual element concentration in a complex [36]. It is necessary to know the exact concentration of an element in water bodies to predict REEs’ toxicity and bioavailability correctly [37]. Inductively coupled plasma mass spectrometry (ICP-MS) and Gas chromatography-inductively coupled plasma-mass spectrometry (GC-ICP-MS), High- Performance Liquid Chromatography (HPLC), non-nuclear, and nuclear analytical techniques (NATs) are some of the best-known techniques used in speciation analysis. However, NATs has unique features, such as precision, accuracy, sensitivity, and reduced matrix effect, compared to non-NATs techniques. In REEs speciation analysis, non-NATs and NATs, such as molecular activation analysis (MAA), proton-induced X-ray emission (PIXE), Mössbauer spectrometry, and synchronous radiation-based analytical techniques are used specifically [38].

In swamps of the USA and groundwater of the Netherlands, speciation analysis significantly demonstrated the dissolved REEs were highly bound with organic matter. The *o*-cresolphthalexon (OCP) used in adsorptive cathodic stripping voltammetry (ACSV) found a strong correlation between pH and dissolved organic carbon (DOC). Moreover, the correlations of REEs with dissolved organic carbon (DOC) increase with increasing atomic number across the REEs series. The REEs speciation and statistical data showed REE^3+^, REE(SO_4_)^+^, REE(CO_3_)^+^, REE(DOC) being the dominant species. Their concentration in the groundwater was affected by pH, which was in approval with successfully aligned statistical data, respectively [39,40].

The group of REEs’ elements exists as free ions, carbonates, and hydroxyl complexes, depending on the pH. Nd is a perfect example of the case [41] showing dependency not only on source rocks but also on prevalent water chemistry. Moreover, carbonate complexes (Ln (CO_3_)_2_^−^ and LnCO_3_^+^) were shown to be the dominant form of REEs in groundwater of pH = 7–9 from California and Nevada regions [42]. Stability constants formations of REEs as carbonate complexes increase with an increasing atomic number across the REEs. Other complex formation with sulfate, phosphate, or hydro-oxide is easier for HREEs than LREEs. The carbonate complexes of HREEs are more stable in solution than the carbonate complexes of LREEs. A filtration experiment performed at low pH with a high concentration of La showed that Yttrium and other REEs exhibit very different behaviors concerning the function of concentration [43].

Speciation of REEs in water bodies is modeled by many research groups [44,45,46,47], but organic matter speciation is still poorly understood compared to inorganic matter. Similarly, the systematic understanding of trace metal complexes in river waters is not fully achieved, and the complication depends on the geographical locations, industrialization, and globalization, weathering, and transport processes [37]. In a study, Donnan dialysis was performed for the speciation of Al, Fe, trace elements, and REEs in 40 water samples collected from 5 coastal lowland acid sulfate soils in northeast Australia, despite the thermodynamic speciation calculation. Here, the proportion of negatively charged humus-REE complexes correlated strongly with one another, except Ce, which was present in the highest concentration. The increment in the complex behavior of LREEs and HREEs was aligned with the pre-observed REEs complex with natural organic matter from humus-rich streams [48], decreasing in complexation with decreasing pH [49]. According to the thermodynamic equilibrium, speciation modeling indicated positively charged sulfate complexes and free cations, are generally dominant for REEs, strongly contrasting with Donnan analysis results [50]. Therefore, the paper’s different results highlighted the requirement for a more detailed study of these elements for more reliability.

## 3. Data Collection for Literature Review

Extensive research was carried out for the compilation of this review paper. A thorough review of the literature was done using various websites like PubMed (http://www.ncbi.nlm.nih.gov/pubmed/), Science Direct (www.sciencedirect.com), and Google Scholar (http://scholar.google.com). Articles related to toxicology in vitro and in vivo were mainly focused on the studies related to aquatic biota and marine ecosystems. The keywords used for searching the relevant data included rare earth elements (REEs) introduction, REEs importance, REEs speciation, REEs toxicity in aquatic life forms (classified separately into prokaryotes, vertebrates, and invertebrates), REEs toxicity in crustaceans, bacteria, and algae, REEs toxicity in rodents, and occupational exposure of REEs.

**Figure 1:**
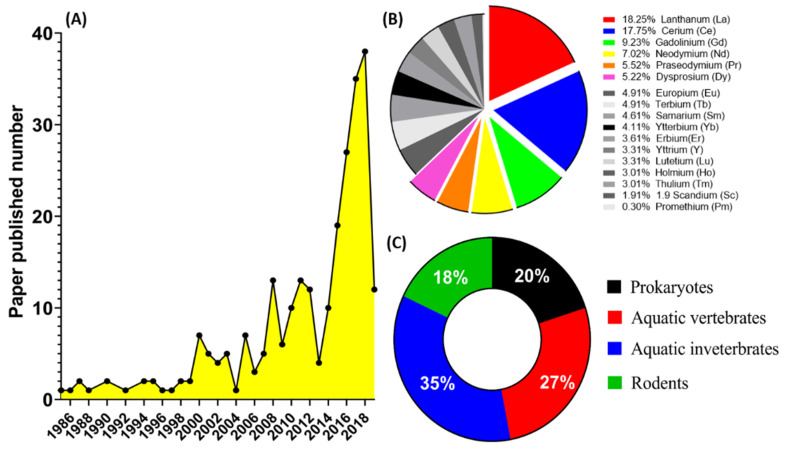
Summary of the rare earth elements (REEs) related research based on published year, REEs species, and toxicity testing animals. (**A**) Number of literatures each year in the eco-toxicity field from 1985–2019. Literature related to rare earth elements was searched in PubMed, Science Direct, and Google Scholar, and the number of literatures was then broken down according to the year. (**B**) Pie chart summarized the number of literatures for each rare earth element (REEs) eco-toxicity test from 1985–2019. The number of literatures was then broken down according to the REEs species. (**C**) Pie chart summarized the number of literatures of model organisms used to analyze REEs toxicity. The model organisms are classified into: prokaryotes, aquatic vertebrates, aquatic invertebrates, and rodents.

The search yielded lots of reports throughout, with very less confidentially established results on REEs toxicity. We counted the literature related to REEs each year from 1985 to March 2019 and found an exponential increase in the number of published papers after 2013 (Figure 1A). Before 2013, the amount of REE-related reports was around 10 per year. By 2018, we noticed that around 40 REEs-related papers being published. Later, REEs-related papers were sorted by REEs species to figure out which element is the most frequently studied. Among 17 REEs, we found La, Ce, Gd, Nd, Pr, and Dy are the top six ranking species since they occupied 2/3 of the total amount of published reports (Figure 1B).

A review of the previously compiled data of research papers on the toxicity of REEs led us to learn various species being used in toxicity analysis, such as prokaryotes, aquatic vertebrates, aquatic invertebrates, rodents, as well as cell lines. These species, as model animals, have been used to study the toxicity of REEs. We can group them into four major categories: prokaryotic (20%), aquatic vertebrates (27%), aquatic invertebrates (35%), and rodent (18%) models (Figure 1C). According to the data, we knew that aquatic invertebrate organisms (e.g., sea urchin, hydra, daphnia, and rotifer) have been more popular REEs toxicity studies in recent years. Figure 1C assigns aquatic vertebrates (zebrafish, rare minnow, and rainbow trout) in second place in terms of neurotoxicity, pharmaceuticals, and morphological testing recognition for studying the parameters beneficial for the mammalian models. Based on the published literature, we found out that no hot spot species being used for REEs’ ecotoxicity test. For example, zebrafish has been widely used for ecotoxicity studies in nanomaterials [51,52], pesticide/herbicide [53,54], heavy metals [55,56,57] as well as solvents [58,59,60]. However, only a few papers have addressed REEs’ acute toxicity using zebrafish embryos [61]. This phenomenon suggests that more zebrafish studies can be done to investigate the potential REEs ecotoxicity in the coming future.

The endpoints studied of the REEs-relevant review paper include: (a) health effects on aquatic animals, rodents, and environment, (b) occasional/occupational exposure effects, (c) dosage response, and (d) effect of physicochemical parameters on REEs. Many papers stated that REEs’ toxicity might depend on the marine ecosystem’s physicochemical properties, such as various trophic levels of rivers, lakes, and estuaries. In contrast, some papers also suggested the dosage, hormetic effects, and size/age dependency of these elements. Thus, it majorly demands more data analysis on several cellular and gene levels to establish reliable results. The citations most relevant to the topic of our review paper have been enlisted in the references.

Furthermore, validating that most industrial effluents and waste products are usually discarded inside the water bodies, it is necessary to understand the toxicity mechanism of REEs in water bodies. We have also discussed the need for extensive study to understand REEs’ role on different concentration levels in marine ecosystems and aquatic organisms. Moreover, many coastal countries rely broadly on the consumption of seafood daily. Therefore, variations or toxicity of REEs aquatic species consumed by humans are worthy of being studied. Some studies suggest the accumulation of some REEs in the human body might result in health hazards after regular exposure.

## 4. Overview of Eco-Toxicity of Lanthanides

Most of the technological applications of REEs have been developed in the last two decades [28]. Thus, they caused a sharp rise in the mining, extraction, refining, and processing of these elements [62,63,64,65]. Most of the relevant reports are concentrated on the impact on aquatic organisms, and Lanthanum (La) is the most frequently studied REE to evaluate the toxic effect to the model organisms. These results highlighted the demand to understand the toxicity mechanisms and bioaccumulation of other REEs [66].

The bioavailability and distribution of lanthanides in the marine environments are dependent on the lanthanide speciation, which is, in turn, influenced by physicochemical parameters of alkalinity, ionic strength, and pH value [67,68]. Lanthanides are fractionated between suspended particles and colloids, and the solution phase in a river or estuarine waters. Thorough observation has suggested that lanthanide’s affinity to colloids and particles is mainly responsible for their distribution and transport of REEs [69]. T. Das et al., demonstrated dissolved lanthanum (La^3+^) represent a small proportion of lanthanum compounds in water and sediment, and they are bioavailable and cause adverse effects in living biological system [70]. La has been reported several effects, particularly on nervous systems, excretory system and smooth muscles [71]. Houda Hanana et al., in 2017, performed a biomarker assessment of lanthanum on *Dreissena polymorpha*. The results revealed La was bioaccumulated in mussels but did not trigger metallothionein (MT) induction. This study also revealed diverse effects on mussels and suggested more research to be performed to understand the exact mechanism of action [71]. Gwyneth Anne MacMillan et al., 2017 demonstrated REEs follow a coherent bioaccumulation pattern for sample tissues, with anomalies for redox-sensitive elements (Ce, Eu). The study showed that terrestrial herbivores, ringed seal, and fish had low total REEs levels in muscle tissue, and accumulation was higher in liver tissues [72]. The bioaccessibility and bioavailability are two important criteria in determining the amount of metal accumulated in aquatic organisms, depending on the shift with environmental conditions. Hence, the accumulation of various REEs can occur through the body surface or food ingestion. Moreover, physiological parameters can influence the metal compounds to be taken up as freely dissolved ions, metal complexes, or particle-bound metals [73]. All this available information must be investigated more thoroughly for specific reasoning and conclusions to establish stable results

## 5. Cumulative Toxicity of REEs, and Malformation in Aquatic Animals

In a study conducted by Romero-Freire et al. [74], a mixture of three lanthanides (Ce, Gd, Lu), representative of the light, medium, and heavy rare earth elements, were used to conduct the toxicity analysis on seven different aquatic species, namely, a symbiotic bacterium (*Aliivibrio fischeri*), microalga (*Raphidocelis subcapitata*), green microalgae (*Chlorella vulgaris*), planktonic rotifer (*Brachionus calyciflorus*), ostracod (*Heterocypris incongruens*), water flea (*Daphnia magna*), and zebrafish (*Danio rerio*). The test results predicted that lanthanide content decreased over time in all test media from the beginning of the test. The major toxic effects of lanthanides were found in *A. fischeri*, *R.* *subcapitata*, and *B.* *calyciflorus*, with an interdependent result between *A.* *fischeri* and *R. subcapitata* in comparison to *B.* *calyciflorus*. Therefore, the variation in the toxicity response from the mixture of lanthanides should be explored further to study individual REEs’ proper interaction with the aquatic organisms for reviewing the interaction level more effectively [74]. On the similar lines, when chloride salts of these three lanthanides Ce, Gd, and Lu were used as CeCl_3_·7H_2_O, GdCl_3_·6H_2_O, LuCl_3_·6H_2_O on a different set of aquatic organisms, *A. fischeri*, microalga (*Pseudokirchneriella subcapitata*), *D. magna*, *H. incongruens*, *B. calyciflorus*, and freshwater polyp (*Hydra attenuate*). The sensitivity of *A. fischeri* and *P. subcapitata* increased with an increasing atomic number in the lanthanides. Other species exhibited a comparable sensitivity to these three tested materials, indicating that some areas’ concentration-dependent effect needs to be more extensively studied over the subject [66]. The mixture and chloride salts Ce, Gd, and Lu in both the studies majorly affect *A. fischeri* and *P. subcapitata* in toxicity and sensitivity terms. However, other species also revealed sensitivity, but no clear results are established. Hence, it renders space for more studies in a mixture of lanthanides to attain specific results.

In another study conducted by Trifuoggi et al. [75], a group of chlorides of 7 REEs (Yttrium (Y), Lanthanum (La), Cerium (Ce), Neodymium (Nd), Samarium (Sm), Europium (Eu) and Gadolinium (Gd) were tested on two sea urchin species: *Sphaerechinus granularis* and *Arbacia lixula*. The developmental defects and cytogenetic anomalies in REEs-exposed embryos/larvae, and decreased fertilization success and offspring damage following sperm exposure were recorded. Results indicated different cytotoxicity patterns for every REE over different species, providing evidence of toxicity scaling for various REEs [75]. Furthermore, in a study of ecotoxicity analysis (LC50 and EC50), *Hydra attenuate* species were exposed to different concentrations of chloride salts of 11 REEs (Y, La, Ce, Pr, Nd, Sm, Gd, Tb, Dy, Er, and Lu) [76]. *Hydra* species are proved to be sensitive to the toxic effect of REEs and heavy metals, which have already been established in earlier work [76,77,78,79,80] as an indicator of metal contamination. In their study, LC50 values ranging from 0.21 to 0.77 mgL^−1^ and EC50 values ranging from 0.02 to 0.27 mgL^−1^ were conveyed, confirming *Hydra’s* sensitivity to REE exposure at different concentrations [76].

Next, when the toxicity of five lanthanides (La, Ce, Pr, Nd, and Gd) was studied on three aquatic microcrustaceans (*Thamnocephalus platyurus*, *Daphnia magna*, and *Heterocypris incongruens*) for 21 days, the results showed that lanthanides were toxic to crustaceans in chronic bioassays. Based on the long-term study on *D. magna*, the paper also suggested considering lanthanides as a uniform group with a similar toxicity mechanism [81].

In the next study model, the biological toxicity of 13 lanthanides was tested over unicellular algae *Skeletonema costatum* to establish the connection between the affluence of lanthanides in seawater and their toxicity over the algae, where all the lanthanides, single or the mixed solution, resulted in same growth inhibition effect on algae. Overlooking the “Harkins rule”, this phenomenon is unique compared to the groups of other elements in the periodic table. The study suggests that the algae might not sufficiently differentiate the chemically identical lanthanides [82]. Another study conducted by Joonas et al. [83], where eight doped and one non-doped rare earth oxide (REO) particles Ce_0.9_Gd_0.1_O_2_, LaFeO_3_, Gd_0.97_CoO_3_, LaCoO_3_, (La_0.5_Sr_0.5_)_0.99_MnO_3_, CeO_2_, Ce_0.8_Pr_0.2_O, (La_0.6_Sr_0.4_)_0.95_CoO_3_, La_2_NiO_4_, respectively, were tested on green algae *Raphidocelis subcapitata* with an OECD201 standard algal growth inhibition assay and 24 h spot test as algal viability assay. Here, the spot analysis demonstrated direct toxicity to algae at 1 mg metal/L, and 72 h inhibition assay value was between 1.2–1.4 mg/L for four REEs salts (Ce, Gd, La, Pr). The inhibition was attributed to nutrient deficiency from the algal growth medium. Interestingly, the paper indicated the adverse effects of Rare Earth Oxides (REOs) particles were partially due to the entrapment of algae within particle agglomerates—the adverse effects of the dissolution of constituent elements doped REO particles and the particle size were excluded. However, the particles’ structure and the varying effects of REO composition might have played a role in the effects. The production rates of these REO particles are negligible compared to other forms of REEs, and there is presumably no acute risk for aquatic unicellular algae. Further, the effects of La^3+^, Ce^3+^, Pr^3+^, Nd^3+^, Gd^3+^, CeO_2_, and eight doped REOs (Ce_0.9_Gd_0.1_O_2_, LaFeO_3_, Gd_0.97_CoO_3_, LaCoO_3_, (La_0.5_Sr_0.5_)_0.99_MnO_3_, CeO_2_, Ce_0.8_Pr_0.2_O, (La_0.6_Sr_0.4_)_0.95_CoO_3_, La_2_NiO_4_) were studied on bacteria *Vibrio fischeri* and protozoa *Tetrahymena thermophila* in parallel with REO-dopant metals (Co^2+^, Fe^3+^, Mn^2+^, Ni^2+^, Sr^2+^) [84]. Here, the highest concentration of REOs tested was 100 mg/L for protozoa in deionized water and 500 mg/L with bacteria in 2% NaCl. The results showed that all studied soluble REEs and Ni^2+^ and Fe^3+^ dopants were toxic to bacteria and protozoa. In this study, the research group concluded that REEs’ toxicity depends on their speciation, which leads to the variation of values obtained from assays performed in different test media. The study indicated that as REEs and REOs form in the environment, insoluble salts, or oxides, they possessed no harm to aquatic bacteria and protozoa.

An old study conducted by Hao et al. (1997) [85], where REEs (La, Gd, and Y) were used as complexes with organic ligands ethylenediaminetetraacetic acid (EDTA), nitrilotriacetic acid (NTA), and citrate (Cit), were calculated in the test medium by Metal Speciation Equilibrium For Surface And Ground Water (MINTEQA) based on aqueous geochemical equilibrium. The influence of the species *Chlorella Vulgaris Beijerinck* on REEs’ bioconcentration processes by algae was also studied. The bioconcentration processes were highly dependent on chemical species in order of RE^3+^ > RE-Cit > RE-NTA > RE-EDTA, where bioconcentration phenomenon was attributed to active sites on the algal surface followed by diffusion into the algal cells. The toxic effect of 12 lanthanides, when investigated over *chlorella autotrophica* demonstrated a 50% reduction in the growth after 96 h culture [86]. This result was also reconfirmed by the chi-square test for dose-dependency, which showed that lanthanide’s biological toxicity on the growth of *chlorella autotrophica* was the same.

Next, studies on the population growth responses of *Tetrahymena shanghaiensis* in exposure to LREE (La, Sm) and HREE (Y, Gd), cellular proliferation assays (cell count, neutral uptake, total protein, and nucleic acid content) were used to evaluate their aquatic toxicity on a 24 h and 96 h assay [87]. The results denoted a dual effect of REEs with stimulating growth at lower concentrations and toxicity at high concentrations, indicating the demand for more study to understand the possible mechanisms of cell growth controlled by REEs. Bio uptake infusion of Eu and Sm when studied on *Chlamydomonas reinhardtii* for 60 min. In the absence and presence of ligands (malic acid and citric acid), bio uptake of Eu or Sm decreased with the increment in the concentration of a competing REE (Eu or Sm), as predicted by biotic ligand model. On the other hand, when the hydrophilic complexes were formed with malic acid as well as citric acid, the bio uptake of Eu was higher as a complex than as a free ion indicating that bio uptake of REEs might vary under the competing REE complexes, which are readily bioavailable [88].

Removal of rare earth elements (REEs) from an acid solution by an algal flagellate, *Euglena gracilis*, was studied by Ishii et al. [89]. Sixteen kinds of REEs were spiked to the solution at a final concentration of 10 μgL^−1^ for each element. *E. gracilis* cells grown in the solution under 12 h light–dark cycles efficiently removed REEs during the 21-day experimental period. Significant removal was observed from days 14 to 21 of incubation. On the last day, REEs’ concentration was less than 0.7 μgL^−1^ except for Sc. The Sc’s concentration was 2.8 ± 0.4 μgL^−1^, suggesting that Sc removal was relatively difficult compared to the other REEs. Among REEs, the same level of removal was observed for light REEs (La–Eu) but not for heavy REEs (Ga–Lu). Heavy REEs removal tends to decrease with an increase in atomic number. The removal of REEs by *E. gracilis* were affected by lighting conditions because *E. gracilis* cells grown in the dark had removed just a small amount of REEs, less than 10% of the total, through day 21 of incubation.

Collectively, REEs can penetrate the membrane, associated with receptors, the blocker of ion-exchange channels, bioaccumulation, specifically targeting Ca^2+^ influx and Ca^2+^ regulated downstream processing has been performed to an extent [90,91]. However, there is a lack of data for defining the safety limits and molecular mechanisms of the activity. The specific parameters of the study have been summarized in Table 2 in the following section.

## 6. Ecotoxicity of Lanthanum (La)

Lanthanum (La), one of the first elements in the lanthanide group, is bioavailable in its trivalent form (La^3+^) and has a high risk of biological effects established in an extensive review [70]. Cytotoxic effects of lanthanum can be defined based on its chemical similarity with alkaline earth elements. During the action, La^3+^ might compete with Ca^2+^ for binding sites in biological systems, inhibiting calcium channels in cell membranes and affecting the work of cells and tissues [56]. In a study to analyze the acute toxicity of La^3+^ on the gill and liver of rare minnow for 21 days, significant changes were observed, establishing the toxic effects of La^3+^ on the gill and liver of rare minnow [95]. A study on the LaCl_3_ exposure to glass eels for 14 days, rapid La accumulation was observed for 3 days, decreasing absorption even after being exposed continually. Acetylcholine esterase (AChE) activity increased, indicating that La^3+^ may inhibit the binding of AChE. A depression in lipid peroxidase activity and catalase inhibition suggest that La^3+^ plays physiological activities and functions as a radical scavenger [96].

Lanthanum oxide nanoparticles (La_2_O_3_ NP) were used to observe acute toxicity on aquatic species *Chlorella* sp. and the *D. magna*. The absence of the toxic effect of La_2_O_3_ NP was confirmed by the growth inhibition assay of *Chlorella* sp. even at a higher concentration (1000 mg/L) after 72 h exposure. Compared with the species, the toxic effects were observed in higher concentrations (e.g., 500 mg/L) for *D. magna*, indicating the need for further risk analysis [97]. A study of the toxicity of lanthanum oxide micro- and nano-sized particles over the gram-positive (*Staphylococcus aureus*) and gram-negative bacteria (*Escherichia coli* and *Pseudomonas aeruginosa*) were carried out [98]. The results indicated that lanthanum oxide showed antimicrobial activity against *S. aureus*, and not on *E. coli* and *P. aeruginosa* was determined by scanning electron microscopy (SEM) and energy-dispersive X-ray spectroscopy (EDS). The antimicrobial activity of La_2_O_3_ NP on gram-positive bacteria was attributed to the interaction of La_2_O_3_ with the gram-positive cell wall of the bacteria [98]. Similarly, when the toxicity mechanism of La^3+^ was detected on *E. coli* in two different concentrations, different results were presented. A low concentration of La^3+^ increased the nutrient absorbance of the cells due to increased cell permeability. A high concentration of La^3+^ increased the accumulation inside the cell, causing a toxic effect on the cell, hence, exhibiting stimulatory and inhibitory effects on the *E. coli* cells [99].

## 7. Ecotoxicity of Cerium (Ce)

Further, in a study conducted by Evseeva et al. [100], when the toxicity of ^232^Th and its stable chemical analogue Ce were analyzed on *Chlorella Vulgaris* after 24 h exposure, EC_50_ and concentration-effect relationship depicted ^232^Th to be more toxic to *chlorella* than Ce, whereas, no observed effect concentration (NOEC) and lowest observed effect concentration (LOEC) of both ^232^Th and Ce were approximately equal. The experiment showed differences in the detoxification pathway for both the elements, pointing towards a more detailed study to understand REEs’ mechanism of action on organisms. In another study conducted by Paoli et al. [101], Ce-containing solutions (0.1 mM, 1 mM, 10 mM, and 100 mM) were tested over lichen *Xanthoria parietina*. The results suggested that bioaccumulation happened through intracellular and extracellular processes, increasing with the increment in Ce concentration and, in turn leading to a decrease in lichen viability. Some study results on *Euglena gracilis* are consecutively established. The cellular uptake mechanism of Ce by *E. gracilis*, when performed by X-ray absorption fine structure (XAFS), demonstrated that cerium was surrounded by 8 N atoms with a bond length of 0.258 nm. The results indicated that the location of REEs in chlorophyll molecules and the intracellular cerium could cross the internal membrane structure of chloroplast; Ce^3+^ in valence form remain unaltered while Ce^4+^ may undergo reduction, ensuring XAFS as an excellent platform to study this further [102]. Moreover, the research group suggested that studies should be performed in vivo to establish concrete results.

## 8. Ecotoxicity of Gadolinium (Gd)

Other lanthanides, such as gadolinium (Gd), were also used to elucidate the toxicity effect. It is a widely used paramagnetic contrast agent for MRI, which leads to adverse effects on human health [103]. Further, in a study, GdCl_3_ and Gd-based MRI contrast agents (Omniscan) were studied on zebra mussels for 28 days with the multi-biomarker approach. The findings suggest that after GdCl_3_ exposure, the mRNA level of MT was modulated. Those of superoxide dismutase (SOD) and cytochrome c-oxidase (CO1) were increased. At the same time, the gene expression of catalase (CAT) and glutathione-s-transferase (GST) were downregulated with no specific effects on lipo-peroxidation (LPO) and genotoxicity. The opposite results displayed by Omniscan depicted the downregulation of SOD, CAT, GST, and CO1 as well as a decrease in LPO and an increase in GST and Cyclooxygenase (COX) activities [104]. Similarly, Gadolinium’s effects on the embryonic development of four sea urchin species, *P. lividus* and *A. lixula* from Europe and *Haliotis tuberculata* and *Centrostephanus rodgersii* from Australia were studied [105]. The work depicted different sensitivities to Gadolinium and dose-dependent skeleton damage with the EC50 values ranging from 56 nM to 132 µM across the tested four species. The study also suggested that the same pollutant can have significantly different toxicity levels on marine organisms, even within the same taxonomic group, which can be misleading in risk assessment.

## 9. Ecotoxicity of Neodymium (Nd)

In a study conducted by Yang et al. [106], the bio uptake of neodymium (Nd) was studied on a green alga, *Chlamydomonas reinhardtii*, to verify the precision of Biotic Ligand Model (BLM) in a controlled study group, the work demonstrated a higher bio-uptake of rare earth metal complexes. However, the group stated that the BLM is not very efficient in foreseeing and anticipating rare earth metals’ uptake. Bioaccumulation of Nd^3+^ in *E. gracilis* was 10 times higher if algae activities were immobilized when the experiment was conducted between living algae and fossil algae [107].

## 10. Ecotoxicity of Yttrium (Y)

In research work done by Aiube et al. [108], Yttrium orthovanadate (YVO_4_) was synthesized in a powder form through combustion (C route) and hydrothermal (H route). On applying YVO_4_ to the fish embryo toxicity test of model organism *D. rerio*, the treated group solution of the C route showed more toxicity than the untreated group with complete mortality by day 6. In contrast, the treated group solution of the H route showed no toxicity enhancement. Such results indicate that microstructural characteristics and synthetic routes are important for producing active materials, but they also impact the effluents’ toxicity. In another study done by Yang et al. [109], the effects of Y^3+^ concentrations on growth characteristics of *Microcystis aeruginosa* FACHB912 and the contents of antioxidant enzymes (SOD, POD, CA, and MDA) were determined. The results depicted altered actions in the growth pattern of *M. aeruginosa* at low concentration (0.10–0.20 mg/L) of Y^3+^ and partial/complete inhibition of their growth under high concentration (0.50–10.00 mg/L). A similar study performed by Wang et al. [110], yttrium, and phosphorus, an essential nutrient element of algae *M. aeruginosa* was investigated. The results depicted an increase in chlorophyll a and soluble protein. The growth of algae is stimulated by yttrium concentration ≤0.3 and 0.2 mg/L phosphorus, while it is inhabited by 0.5 and 1.0 mg/L Yttrium. The activity of superoxide dismutase (SOD) of algae also increases with addition of yttrium dose (0–0.3 mg/L) when phosphorus dose is 0.2 mg/L. The malondialdehyde (MDA) increased with time and concentration of yttrium. However, the addition of 0.2 mg/L Phosphorus weakens the accumulation of MDA. The research group revealed a relationship between heavy REEs ions and heavy metal ions acting on *M. aeruginosa* growth and physiological characteristics. It demands further research on the other model organisms to understand the underlying mechanism.

## 11. Ecotoxicity of Praseodymium (Pr)

When *Praseodymium*’s effect was studied on the physiological, biochemical, and ultrastructural responses of *Spirodela polyrhiza* at a concentration range of 0–60 µM for 20 days, the obtained results indicate an effect on species in a concentration-dependent manner. A significant increase in cell death was observed with a reduction in photosystem II activity, indicating the presence of Pr-inducing oxidative stress. A significant increase in cell death was observed in Pr-treated plants. *S. polyrhiza* was observed to combat Pr induced oxidative injury by activating various enzymatic and non-enzymatic antioxidants [111].

## 12. Ecotoxicity of Samarium (Sm)

The Sm speciation in the presence of natural organic matter (NOM) and its bioavailability to *Chlamydomonas reinhardtii* were determined. The short-term bio uptake experiments were performed in four type NOM Suwannee river fulvic acids, Suwannee river humic acids, Pahokee peat fulvic acids, and Luther marsh dissolved organic matter isolates. The result revealed that even a small amount of NOM (0.5 mg C L^−1^) significantly decreased Sm internationalization fluxes (10 times). It was shown that free Sm was a better predictor of Sm internalization than either total concentrations or the free ion concentrations obtained using thermodynamic modeling [112].

## 13. Ecotoxicity of Dysprosium (Dy)

Dysprosium’s effect on freshwater invertebrates of *Daphnia pulex* and *Hyalella Azteca* to understand toxicity modifying influence of Ca, Na, Mg, pH, and dissolved organic matter (DOM). Acute toxicity tests were performed on <24 h old neonates for 48 h in *D. pulex* and 2–9 day old offspring for 96 h tests with *Hyalella*. The dissolved Dy concentrations were lower than total (unfiltered) Dy indicating precipitation prominently at high concentrations. *Hyalella* demonstrated 1.4 times more sensitive than *Daphnia* in terms of Dy toxicity. Ca and Na addition provided significant protection to *Hyalella* against Dy toxicity. Overall, this study contributed data to site-specific water quality for Dy toxicity [113]. In a study to assess the effect of Dy on a microcosm, it was described that other coexisting species modified the effect of Dy on one species in the microcosm [114]. The study pointed out that further studies are required to analyze Dy toxicity on aquatic microbial communities.

## 14. Ecotoxicity of Holmium (Ho)

The microcalorimetric technique was used to analyze the biological effect of Ho^3+^ on *Halobacterium halobium*. Ho^3+^ caused a decrease in maximum heat production and growth rate constants. Comparing the results of thermogenic curves and Transmission electron microscopy (TEM), it was clarified that the metabolic mechanism of *H. halobium* R1 growth was changed with the addition of Ho^3+^ [115]. The specifications of the study are shown in Table 3 in the following section.

## 15. Discussion and Future Research Direction

As prospective contaminants, REEs can enter the aquatic ecosystem through many sources, such as weathering, transportation, and sewage treatment plants. The natural environment of the water affects the speciation of REEs in water bodies to a greater extent. Physicochemical characteristics of organisms influence their bioavailability, bioaccumulation, and sorption of REEs. Considering the increasing demand for REEs, it is vital to acquire data related to REEs-associated biological effects. These REEs’ safety concentration has not yet been established, but some studies with certain elements have described them efficiently. The sediments in the water bodies can carry and transport these REEs in a specific manner, and thus, REEs are also considered major pollutants. It is vital for mineralogical and geological research to focus on the deposition, extraction, mobility, interaction with colloids, transportation, and REEs’ sorption to ensure some concrete results in the near future [38]. REEs have been shown to have crossed the intracellular membranes, generated reactive oxygen species (ROS), lipid peroxidation, and modulation of anti-oxidation activities. However, specific end-points, such as cell growth, membrane structure, and some specific functions need to be addressed more aggressively and prominently to establish some safe limits of usage for plants, animals, and humans [28].

The currently thriving related literature to REEs toxicity to aquatic life forms is mostly confined to certain elements (La, Gd, Nd, and Y), thus, leaving scarce information about other REEs and their colloids. The related studies and evaluations are therefore required to handle the rise in the usage of all kinds of REEs to efficiently set up the safety limits and ensure no harmful effects on the environment or health of existing species. The research funding and speciation protocols and techniques should also be provided to perform more detailed studies in this respect. Thus, several REEs’ information remains relatively scarce, notwithstanding their growing industrial utilization and hence, environmental spread and human exposures. This gap, along with several open questions in REEs health effects shall be filled in future investigations.

The ever-growing market of REEs has caused a high toxicity concern, which requires further analysis to establish the toxicity effects and safety parameters. REEs’ ecotoxicity issues have also increased in the last two decades, compared to other trace elements. However, there is a scarcity of information on the toxicity and accumulation of these metals. The data we acquired for this review paper is based only on a relatively small pool of references related to REEs ecotoxicity, which can be considered primary data, bound to change when further data is acquired to be processed and published. The aquatic environment forms an effective sink of REEs, usually dissolved phases, and other trace elements. They are adsorbed in sediments as well as other aquatic flora and fauna, which can be exposed to humans through multiple intake routes of iatrogenic, inhalation, and occupational exposure. This review briefly describes REES’ relevant research, leaving a significant gap in knowledge to the toxicity effect in a variety of environments and other categories. Therefore, there is a need to fill the existing information gap and establish an environment fingerprint for utilizing REEs in terms of safety.

**Figure 2:**
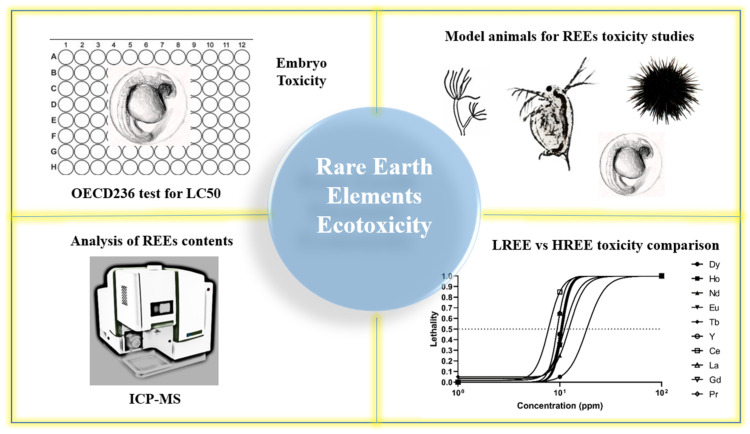
Summary of the fish and other simple animal models that can be used to study rare earth elements (REEs) toxicity. (Upper left panel) zebrafish is suitable to conduct high throughput toxicity assay due to its small size and transparent out looking. (Upper right panel) other animal models like hydra, daphnids, and sea urchin also can be used for the REE toxicity study. (Bottom left panel) inductively coupled plasma mass spectrometry (ICP-MS) provides high sensitivity to detect the bioaccumulation and distribution of REEs in the animal body. (Bottom right panel) the acute toxicity of REEs can be easily measured in zebrafish by calculating the 96-h LC50 value.

Not many researchers pay attention to REEs contamination hazards, which is exposed to fauna and flora and human life. In the above-mentioned direction of work, the research groups should be taken up to enhance the knowledge and enrich REEs’ toxicity information to different life forms. In our laboratory, we prepare to take a model organism, zebrafish (*D. rerio*), as a platform to perform the comprehensive measurements of multiple behavior endpoints and biochemical analysis to evaluate toxicity parameters to contribute data in this research, taking it a step further (Figure 2). There is a need for the research groups to come up with solutions for resource limitations to test various REEs on model organisms employing alternative testing methods for better understanding the toxicity caused by REEs even on mammalian models. In comparison to other animal models, zebrafishes are relatively inexpensive to maintain. They can produce numerous fertilized eggs by natural mating in the laboratory, grown in 96 well assay plates individually. A fast embryonic developmental process, transparent embryos allowing easy visualization of effects being caused in the embryos with the help of a microscope, and informative genetic, genomic materials and tools to perform forward and reversed gene function manipulation can also be done. These combined advantages make zebrafish a popular vertebrate model for performing ecotoxicity tests in recent decades [116,117,118,119,120,121]. It will also be an excellent model for testing the potential ecotoxicity of REEs in the coming future.

## Figures and Tables

**Table 1:** Summary of the industry usage of rare earth elements (REEs).

Industry Usages	Rare Earth Elements (REEs)
Cordless power tools	Praseodymium (Pr), Neodymium (Nd), Terbium (Tb), Dysprosium (Dy)
Optics	Yttrium (Y), Europium (Eu), Terbium (Tb)
Energy-saving light bulbs	Yttrium (Y), Europium (Eu)
Liquid-Crystal Display (LCD)/Plasma Display Panel (PDP) screen displays	Yttrium (Y), Cerium (Ce), Europium (Eu), Terbium (Tb)
Wind turbines	Praseodymium (Pr), Neodymium (Nd), Dysprosium (Dy)
Hybrid vehicles	Praseodymium (Pr), Neodymium (Nd), Samarium (Sm), Terbium (Tb), Dysprosium (Dy)
Digital camera lenses, Rechargeable batteries	Lanthanum (La), Cerium (Ce)
Speakers, earphones	Praseodymium (Pr), Neodymium (Nd), Gadolinium (Gd)
Magnets	Praseodymium (Pr), Neodymium (Nd), Gadolinium (Gd), Terbium (Tb), Dysprosium (Dy)
Defense, guidance, and control	Praseodymium (Pr), Neodymium (Nd), Samarium (Sm), Terbium (Tb), Dysprosium (Dy)
IPod, CD/DVD, smartphone	Lanthanum (La), Cerium (Ce), Praseodymium (Pr), Neodymium (Nd)
Agriculture	Lanthanum (La), Cerium (Ce)
Laser, Light-Emitting Diode (LED), fluorescent lamps	Lanthanum (La), Cerium (Ce), Europium (Eu), Terbium (Tb), Yttrium (Y)
Health	Praseodymium (Pr), Neodymium (Nd), Gadolinium (Gd), Terbium (Tb), Dysprosium (Dy)
Petroleum refining, automotive catalysts, diesel addictive	Lanthanum (La), Cerium (Ce), Praseodymium (Pr), Neodymium (Nd)
Colorants, fuel cells, capacitors, sensors, semiconductors	Lanthanum (La), Cerium (Ce), Praseodymium (Pr), Neodymium (Nd), Gadolinium (Gd), Erbium (Er), Holmium (Ho)

**Table 2:** Summary of the cumulative effects of rare earth elements (REEs) on aquatic organisms.

REEs	Model Organisms	Concentration	Time Duration	Toxicity Analysis	LC50/EC50	Reference
Ce, Gd, and Lu	*A. fischeri*, *R. subcapitata*, *C. vulgaris*, *B. calyciflorus*, *H. incongruens*, *Daphnia magna*, and *Danio rerio*	CE1, CE2, CE3, Gd1, Gd2, Gd3, Lu1, Lu2, Lu3, Dual Salt mixture (A, B, C) 12 Treatments in different Concentrations (μg/L)	MIT 0.5 h LUT 4 h ALT 72 h ROT 48 h OST 144 h DAT 48 h FET 96 h	Calculated from t = oh to t = xh different results, according to concentration and time duration	NA	[74]
CeCl_3_·7H_2_O, GdCl_3_·6H_2_O, LuCl_3_·6H_2_O	*A. fischeri*, *Pseudokirchneriella subcapitata*, *D. magna*, *H. incongruens*, *B. calyciflorus*, and *Hydra attenuate*	100, 200, 400, 800, 1600, 3200, and 6400 mg/L	48, 72, 96 h and 6 days	Considering the toxicity of the three lanthanides studied, crustaceans were the least sensitive species and rotifer, and cnidarians were the most sensitive ones	LC50 values for *D. magna* and *H. incongruens* organisms, and EC50 for growth in *H. incongruens*, are anticipated to be greater than 6400 mg/L	[66]
Yttrium (Y), La, Ce, Nd, Sm, Eu, Gd	Two Sea urchin species of *Sphaerechinus granularis* and *Arbacia lixula*	10^−7^ to 10^−4^ M	72 hpf, 5 hpf,	Embryotoxicity and offspring damage	NA	[75]
Y, La, Ce, Pr, Nd, Sm, Gd, Tb, Dy, Er, Lu	Freshwater cnidarian *Hydra attenuata*	1 g/L stock solution—in *Hydra* medium, 10 mg/L for subsequent bioanalysis	96 h	Hydra morphology based on the Welby scale	y-0.22, La-0.21, Ce-0.33, Pr-0.56, Nd-0.31, Sm-0.77, Gd-0.52, Tb-0.70, Dy-0.69, Er-0.40, Lu-0.29 mg/L	[76]
La, Ce, Pr, Nd, and Gd	*Thamnocephalus platyurus*, *Daphnia magna*, and *Heterocypris incongruens*	0.01, 0.1, 0.25, 0.5 and 1.0 mg Ln/L	21 days reproduction tests	Lanthanides were very toxic to crustaceans showing potential hazards for aquatic ecosystems.	0.3–0.5 mg Ln/L	[81]
LaCl_3_, Ce(N)_3_)_3_, NdCl_3_, SmCl_3_, Eu(NO_3_)_3_, Gd(NO_3_)_3_, TbCl_3_, DyCl_3_, HoCl_3_, Er(NO_3_)_3_, TmCl_3_, YbCl_3_, LuCl_3_, ScCl_3_, Y(NO_3_)_3_	*Skeletonema costatum*	5, 10, 20, 30, and 40 lmol L^−1^ Yttrium were 20, 30, 40, 50, and 60 lmol L^−1^	72 h	There is no difference in the LLG and HLG on inhibition of algae cells,	96-EC50 of La, Y and Sc on *S. costatum* was 29.19 lmol L^−1^, 43.21 lmol L^−1^ and 21.88 lmol L^−1^	[82]
CGO, LFO, GCO, LCO, LSM, CEO, CPO, LSC, LNO	*Raphidocelis subcapitata*	1–100 mg/L	72 h	Two main mechanisms of inhibition by REEs: Nutrient removal from the algal medium Agglomeration of particles around algal cells	EC50 1 to 98 mg/L	[83]
CGO, LFO, GCO, LCO, LSM, CEO, CPO, LSC, LNO	*Vibrio fischeri* and *Tetrahymena thermophila*	1, 10 and 100 mg/L	24 h	Most REOs produced reactive oxygen species; all studied soluble REEs were toxic to bacteria. Dopant metals (Ni^2+^, Fe^3+^) proved toxic, no toxicity of REOs to protozoa and bacteria was observed except for La_2_NiO_4_	*Bacteria* EC_50_ 3.5–21 mg metal/L *Protozoa* EC_50_ 28–42 mg/L	[84]
La, Gd, and Y with ligands EDTA, NTA, and Cit.	*Chlorella Vulgarize Beijerinck*	1.0 mg/L	96 h	Adding organic ligands that can form the RE-Organic species complex led to a great reduction of the REEs’ bio-concentration in *algae*.	NA	[85]
La, Sm, Y, and Gd	*Tetrahymena shanghaiensis*	0.125, 0.25, 0.5, 1.0, 2.0, and 4.0 mM	24 and 96 h	24- and 96-h assays suggest a dual effect of REEs on *T. shanghaiensis* of the stimulated growth at low concentrations and the toxicity at higher concentrations	NA	[87]
Eu and Sm	*Chlamydomonas reinhardtii*	1×10^−9^–1×10^−5^ M	60 min	REE was likely to share a common bio uptake pathway; the bio uptake of a one REE was reduced when another was present, and REE complexes were bioavailable.	NA	[88]
Sm and Y	*Dreissena polymorpha* (freshwater Mussel)	10, 50, 250 and 1250 µg/L	28 days	Y more toxic than Sm, (further toxicity analysis required).	NA	[92]
La, Yb	*Danio rerio* embryos	0, 0.01, 0.1, 0.3, 0.5 and 1.0 mM	Time points—8, 24, 32, 48–60, 72, and 96 h	Yb more toxic than La (Some other Malformations Indicated Dose dependency)	Median LC_50_ La^3+^ and Yb^3+^ were 0.603 and 0.268 mmol/L	[61]
Scandium (Sc), Y, La, Ce, Pr, Nd, Sm, Eu, Gd, Tb, Dy, Ho, Er, Tm, Yb, and Lu	*Euglena gracilis*	10 g/L	7 days	Removal of REEs by *E. gracilis* from acidic solution was demonstrated. This ability of *E. gracilis* suggests a potential use in wastewater remediation.	NA	[89]
Dy, Ho, Er, Yb and Lu, Ce(III)	*Paracentrotus lividus* and *Arbacia lixula* (Embryo and Sperm)	Embryos exposure (10^−7^ to 10^−5^ M), 50-µL sperm pellet10^−5^ to 10^−4^ M	Embryos—10 min—72 hpf. Sperm—1 h in 30 mL FSW Offspring, residual HREE concentrations of 5 × 10^−8^ and 5 × 10^−7^ M	Different toxicity of different HREEs in concentration 10^−6^ M for embryos, to 10^−5^–10^−4^ M for sperm	Comparable effects of REEs according to Different endpoints in the paper.	[93]
Y, La, Ce, Nd, Sm, Eu and Gd	Sea urchin (*Paracentrotus lividus*) (embryo and sperm)	10^8^ to 10^4^ M	10 min—72 hpf	Nd (III) and Sm (III) resulted in relatively lesser toxicity in the tested endpoints	NA	[94]
CeCl_3_–7H_2_O GdCl_3−_6H_2_O LuCl_3-_6H_2_O	*Aliivibrio fischeri* *Pseudokirchneriella subcapitata* *Daphnia magna* *Heterocypris incongruens*, *Brachionus calyciflorus* *Hydra attenuata*	100, 200, 400, 800, 1600, 3200, and 6400 μg/L	Different Time periods for all different test	Ecotoxicity increased with an increase in atomic number in bacteria and algae No general pattern established	NA	[66]

**Table 3:** The summary of the effects of individual rare earth elements (REE).

REE	Model Organisms	Concentration	Time Duration	Toxicity Analysis	LC_50_/EC_50_	Ref.
Lanthanum (La (III))	*Rare minnow* (*Gobiocypris rarus*)	(0.0, 0.1, 0.5, 1.0, 5.0, 10.0, and 40.0 mg/L)	96 h	Severe acute Toxic effects of La (III) on organisms after 21 d of exposure, abnormal dose-dependent behavior	LC_50_ of La (III)at 96 h (1.92 mg/L)	[95]
LaCl_3_	*Anguilla Anguilla*	120 ng/L	14 days	A significant increase in AChE activity, depression in lipid peroxidation and CAT inhibition	NA	[96]
La_2_O_3_ NP	*Chlorella* sp. and *Daphnia magna*	10, 50, 100, 250, 500, and 1000 mg/L	2, 24, 72 h	Absence of toxic effects of La_2_O_3_ NP on *Chlorella* sp., even at higher concentrations (1000 mg/L) after 72 h exposure. No significant toxic effects were observed on *D. magna* at conc. of 250 mg/L or less, and considerable toxic effects were noted in higher concentrations	EC50-500 mg L^−1^; lethal dose LD50 1000 mg/L	[97]
La_2_O_3_	*S. aureus*, *E. coli*, and *P. aeruginosa*	100 µL	24 h	Antimicrobial activity against *Staphylococcus aureus*, but not against *Escherichia coli* and *Pseudomonas aeruginosa*	NA	[98]
La	*Daphnia carinata*	100, 200, 400, 600, 800, and 1000 μg La/L. In Tap Water, *Daphnia* Water, Hard water (ASTM)	24–48 h	Acute and chronic toxicity	NA	[99]
Thorium (^232^Th) and stable chemical analogue Ce	*Chlorella vulgaris*	0.001–28.013 µM ^232^Th and 0.036–71.367 µM Ce	24 h	^232^Th was more toxic to *Ch. vulgaris* after a 24-h exposure than its nonradioactive chemical analogue Ce. Detoxification pathways of Ce (III) in *Ch. vulgaris* differ from those for ^232^Th	NA	[100]
Ce	*Xanthoria parietina*	0.1 mM, 1 mM, 10 mM, and 100 mM	24 h	Ce bioaccumulation, both extracellularly and intracellularly, caused acute toxicity, evident as decreased sample viability, decrease in photosynthetic performance, and changes in the ultrastructure of the lichen *X. parietina*	NA	[101]
CeCl_3_, Ce(SO_4_)_2_	*Euglena gracilis*	3.3 µg/mL	2 h	Intracellular lanthanide was able to pass through the chloroplast’s internal membrane system until the replacement of Mg in chlorophyll molecules	NA	[102]
GdCl_3_, gadolinium-based MRI contrast agent (Omniscan)	*D. polymorpha*	10, 50, 250, and 1250 mg/L	28 days	Gd accumulated in zebra tissue after GdCl3 exposure was highly correlated to SOD, CAT, GST, and CO1 gene expressions and also to COX. The major factors affected by Omniscan were GST gene expression, SOD, CO1 and LPO	NA	[71]
Gd	*Paracentrotus lividus* and *Arbacia lixula* *Heliocidaris tuberculata* and *Centrostephanus rodgersii*	1 to 125 µM	48 hpf	All four species different had different sensitivity to Gd, but the effect of this agent on larval phenotype was similar	EC50 56 nM to 132 µM	[105]
Nd	*Chlamydomonas reinhardtii*	1 × 10^−9^ to 1 × 10^−5^ M	1, 15, 30, 45 and 60 min	A Michaelis–Menten equation described Nd bio uptake with an affinity constant, K_Nd_, of 10^6.8^ M^−1^ and a maximum internalization flux of J_max_ = 1.70 × 10^−14^ mol cm^−2^ s^−1^	NA	[106]
Nd^3+^	*Euglena gracilis*	50 mg/L	30 min	The bioaccumulation efficiency of the rare earth metal neodymium by the green algae species *E. gracilis* can be ten times higher if the algae activity is stopped by immobilization	NA	[107]
YVO_4_	*D. rerio* embryos	18.75 mg	6 days	Inhibition of egg hatching and mortality of *D. rerio* embryos after hatching	NA	[108]
Y^3+^	*Microcystis aeruginosa*	Y^3+^ 0.00, 0.10, 0.20, 0.50, 1.00, 2.00, 5.00, 10.00 mg/L, and (1.00 mg/L of Pb^2+^ constant)	16 days	0.10–0.50 mg/L Y^3+^ could stimulate the growth of *M. aeruginosa* under lead stress. Low concentration of Y^3+^ would induce less ROS generation and oxidative stress under Pb stress evident from activities of SOD, POD, CAT, and MDA	NA	[109]
Y	*Microcystis aeruginosa*	Yttrium 0.0, 0.1, 0.3, 0.5, 1.0 mg/L, and 0.02, 0.2 mg/L phosphorus	20 days	0.1 and 0.3 mg/L yttrium doses promote the growth of *M. aeruginosa*, while the growth will be inhibited by 0.5 and 1.0 mg/L yttrium. The effects, which are caused by yttrium are different when algae are exposed to different doses of phosphorus	NA	[110]
Pr	*Spirodela polyrhiza*	0 to 60 μM	20 days	Significant increases in malondialdehyde (MDA), and decreases in photosynthetic pigment, soluble protein, and unsaturated fatty acids showed that Pr induced oxidative stress. Inhibitory effects on photosystem II and the degradation of the reaction center proteins D1 and D2 were revealed by chlorophyll *a* fluorescence transients, immunoblotting, and damage to chloroplast ultrastructure	NA	[111]
Sm^3+^	Green alga *Chlamydomonas reinhardtii*	10^−9^ and 10^−5^ M	1, 5, 10, and 15 min	Toxicity is likely to be dependent on free ion concentration	NA	[112]
Dy	*Daphnia* pulex and *Hyalella azteca*	500, 500, 125, 25, and 5 M	*Hyalella* (96 h) *Daphnia* (48 h)	*Hyalella* to be 1.4 times more sensitive than *Daphnia*. The addition of Ca/Na protects *Hyalella* against Dy toxicity. Low pH was associated with a reduction in toxicity	Acute Dy exposure to *D. pulex* LC50 = 3.0 µM), *H. Azteca* LC50 = 2.1 µM)	[113]
Dy	Aquatic microbial microcosm consisting of flagellate algae—*Euglena Gracilis, Ciliate protozoa- Tetrahymena thermophila, Bacteria—E.coli*	50, 100, 180, 300, 560, 1000 µM	10 days–120 days, in accordance with the microbial microcosm	Toxicity of Dy was mitigated in microcosm compared to pure culture system for all different species of microcosm	NA	[114]
Ho^3+^	*Halobacterium halobium*	10, 20, 40, 60, 80, and 100 μg/mL	13,000 min	Values of Pm and k are linked to the concentration of Ho^3+^, Ho^3+^ causes a decrease of the maximum heat production and growth rate constants	NA	[115]

## Abbreviations

AChE	Acetylcholine Esterase
ACSV	Adsorptive Cathodic Stripping Voltammetry
BLM	Biotic Ligand Model
CAT	Catalase
Ce	Cerium
Cit	Citrate
C route	Combustion
COX	Cyclooxygenase
CO1	Cytochrome c-oxidase
DOC	Dissolved Organic Carbon
Dy	Dysprosium
EDS	Energy-Dispersive X-ray Spectroscopy
Er	Erbium
EDTA	Ethylenediaminetetraacetic
Eu	Europium
Gd	Gadolinium
GC-ICP-MS	Gas Chromatography-Inductively Coupled Plasma-Mass Spectrometry
GFR	Glomerular Filtration Rate
GST	Glutathione-s-transferase
HPLC	High- Performance Liquid Chromatography
HREEs	High Rare Earth Elements
Ho	Holmium
H route	Hydrothermal
ICP-MS	Inductively Coupled Plasma Mass Spectrometry
La	Lanthanum
LREEs	Light Rare Earth Elements
LPO	Lipid peroxidation
LOEC	Lowest Observed Effect Concentration
Lu	Lutetium
MRI	Magnetic Resonance Imaging
MINTEQA	Metal Speciation Equilibrium For Surface And Ground Water
MT	Metallothionein
MAA	Molecular Activation Analysis
NOM	Natural organic matter
NSF	Nephrogenic Systemic Fibrosis
Nd	Neodymium
NTA	Nitrilotriacetic acid
NOEC	No Observed Effect Concentration
NATs	Nuclear Analytical Techniques
OCP	*O*-cresolphthalexon
Pr	Praseodymium
Pm	Promethium
PIXE	Proton-Induced X-ray Emission
REEs	Rare Earth Elements
REOs	Rare Earth Oxides
Sm	Samarium
SEM	Scanning Electron Microscopy
SOD	Superoxide Dismutase
Tb	Terbium
Tm	Thulium
USGS	United States Geological Survey
XAFS	X-ray Absorption Fine Structure
Yb	Ytterbium
